# Supplementary material for: Tunable entangled photon-pair generation in a liquid crystal
Source: Nature. 2024 Jun 12;631(8020):294–9. doi: 10.1038/s41586-024-07543-5 (PMC11236711; doi:10.1038/s41586-024-07543-5)
Supplement: Supplementary file 1 — This Supplementary Information file contains the following 5 sections: (1) Refractive index measurement; (2) Sample characterization via second-harmonic generation; (3) Possible effects of the pump beam on the sample and other nonlinear effects; (4) Maximum likelihood method; and (5) References. [file 41586_2024_7543_MOESM1_ESM.pdf]

nature portfolio

https://doi.org/10.1038/s41586-024-07543-5

Supplementary information

Tunable entangled photon-pair generation in a liquid crystal

In the format provided by the authors and unedited

Supplementary Materials for Tuneable entangled photon pair generation in a liquid crystal

Vitaliy Sultanov,† Aljaž Kavčič,† Emmanouil Kokkinakis, Nerea Sebastián, Maria V. Chekhova,* Matjaž Humar

† These authors contributed equally
*To whom correspondence should be addressed; E-mail: maria.chekhova@mpl.mpg.de.

1 REFRACTIVE INDEX MEASUREMENT

We measured the material's refractive index and birefringence with an Abbe refractometer. The material was dropcasted on the imaging slide of the refractometer without any aligning layer and left to stabilize on the refractometer for one day before the measurements. We measured the refractive index as a function of wavelength using a set of narrow bandpass filters with $10\,\mathrm{nm}$ bandwidth. The ordinary value of the refractive index was estimated from the lower index boundary line, while the extraordinary value was estimated for the orthogonal incoming light polarization from the higher index boundary gradient. Additionally, the birefringence of the sample was independently measured via the wedge cell method, which showed a consistent result with the birefringence calculated from the measured refractive index values. We fitted the measured values of the refractive index with a two-term Cauchy model $n(\lambda) = A + B/\lambda^2$ to extrapolate the refractive index dispersion to the infrared region. $A = 1.663$, $B = 10\,256\,\mathrm{nm}^2$ for extraordinary refractive index and $A = 1.474$, $B = 874\,\mathrm{nm}^2$ for ordinary refractive index.

2 SAMPLE CHARACTERIZATION VIA SECOND-HARMONIC GENERATION

We characterize the second-order nonlinearity of LC by measuring second-harmonic generation (SHG) in one of the samples and comparing it with the SHG in a known material (Extended Data Fig. 3). For comparison, we took a thin layer of 5% magnesium-doped lithium niobate (5% $MgO{:}LiNbO_3$, LN) with a thickness of $7\,\mu\mathrm{m}$. The sample under investigation was a $7\,\mu\mathrm{m}$-thick cell of FNLC-1751 with no molecular twist to avoid polarization transformation effects

on SHG. Since the nonlinear tensor of LN is well known, we retrieved information about the nonlinear tensor of LC by comparing the SHG efficiency in LN and LC measured under the same experimental conditions.

As a pump, we used light generated at 1370 nm from a homemade optical parametric generator (OPG) pumped at 532 nm (20 ps pulse duration). With a set of a polarizer and a half-wave plate (HWP), installed both before and after the sample, we measured SHG in LN and LC as a function of the pump polarization and the detected second-harmonic polarization. We could retrieve the relative values of the nonlinear tensor for LC compared to LN from the obtained dependencies.

The second-order nonlinearity of any material is generally described by the $\hat{\chi}^{(2)}$ or d tensor. The latter has the form [1]

$$\hat{d} = \begin{pmatrix} d_{11} & d_{12} & d_{13} & d_{14} & d_{15} & d_{16} \\ d_{21} & d_{22} & d_{23} & d_{24} & d_{25} & d_{26} \\ d_{31} & d_{32} & d_{33} & d_{34} & d_{35} & d_{36} \end{pmatrix}. \tag{S1}$$

Omitting the geometrical factors, phase-matching, and constants, the relation between the second-harmonic electric field $\vec{E}_{SH}$ and the pump electric field $\vec{E}$ is [2]

$$\begin{pmatrix} E_{\parallel} \\ E_o \\ E_e \end{pmatrix}_{SHG} = \begin{pmatrix} d_{11} & d_{12} & d_{13} & d_{14} & d_{15} & d_{16} \\ d_{21} & d_{22} & d_{23} & d_{24} & d_{25} & d_{26} \\ d_{31} & d_{32} & d_{33} & d_{34} & d_{35} & d_{36} \end{pmatrix} \begin{pmatrix} E_{\parallel}^2 \\ E_o^2 \\ E_e^2 \\ 2E_oE_e \\ 2E_{\parallel}E_e \\ 2E_{\parallel}E_o \end{pmatrix}_{PUMP}, \tag{S2}$$

where indices e, o, $\parallel$ define the components of the electric field along the extraordinary axis, ordinary axis, and longitudinal component of the field, respectively. While the latter is usually equal to zero, the first two components depend on the pump polarization with respect to the orientation of the crystal axes. For LN, the d tensor has the form [1]

$$\hat{d}_{LN} = \begin{pmatrix} 0 & 0 & 0 & 0 & d_{31} & -d_{22} \\ -d_{22} & d_{22} & 0 & d_{31} & 0 & 0 \\ d_{31} & d_{31} & d_{33} & 0 & 0 & 0 \end{pmatrix}, \tag{S3}$$

where $d_{22} \approx 2.1 \frac{pm}{V}$, $d_{31} \approx -4.3 \frac{pm}{V}$, and $d_{33} \approx -34 \frac{pm}{V}$ [3]. If we place LN with the extraordinary axis being horizontally oriented, then the horizontal and vertical components of the generated second harmonic field $E_H^{(SHG)}$ and $E_V^{(SHG)}$ are

$$E_H^{(SHG)} = d_{33}^{(LN)} E_H^2 + d_{31}^{(LN)} E_V^2, \tag{S4}$$

$$E_V^{(SHG)} = d_{22}^{(LN)} E_V^2 + 2d_{31}^{(LN)} E_H E_V, \tag{S5}$$

where $E_H = \cos(\theta_P) E_p$ and $E_V = \sin(\theta_P) E_p$ are the horizontal and vertical projections of the pump field, with θ_P being the angle between the pump polarization plane and the extraordinary

axis of LN. The total detected SHG intensity is the function of the analyzer orientation given by angle θ_A between the horizontal orientation and the transmitted second-harmonic polarization,

$$I_{SHG} \propto \left| E_H^{(SHG)} \cos\theta_A + E_V^{(SHG)} \sin\theta_A \right|^2 . \tag{S6}$$

We use this equation to fit the measurement results and retrieve the values of the second-order nonlinear tensor of LC. In the experiment, the d tensor of LC is defined with the extraordinary axis oriented vertically along the molecular orientation. Without any longitudinal fields involved and with no sample rotation, it is possible to retrieve 6 components of the d tensor, d_{22}, d_{23}, d_{24}, d_{32}, d_{33}, d_{34}:

$$E_H^{(SHG)} = d_{22}^{(LC)} E_H^2 + d_{23}^{(LC)} E_V^2 + 2d_{24}^{(LC)} E_H E_V, \tag{S7}$$

$$E_V^{(SHG)} = d_{32}^{(LC)} E_H^2 + d_{33}^{(LC)} E_V^2 + 2d_{34}^{(LC)} E_H E_V. \tag{S8}$$

although, due to uniaxial symmetry, there are complementary components with the same values. To properly compare the SHG efficiencies in LN and LC from the measured intensities, we also considered the difference in the refractive index of two materials [2].

First, we measure the second harmonic from LN and LC with the fixed pump polarization horizontally or vertically (panel a in Extended Data Fig. 3). For LN with the crystal axis oriented horizontally, the SHG efficiency must follow

$$\eta_{SHG} = \frac{P_{SH}}{P_P^2} = \frac{(d_{33}\cos\phi_A)^2}{n_{LN}^2(\omega_P) n_{LN}(2\omega_P)}, \tag{S9}$$

where indices SH, P, and A stand for second harmonic, pump, and analyzer, respectively. We used this equation to fit the measured SHG efficiency in LN from the horizontally polarized pump (red curve in Extended Data Fig. 3a) to retrieve the relative value of the d_{33} component of the LN nonlinear tensor for the further comparison with the SHG efficiency in LC, which is 2.4 ± 0.03 in arbitrary units. Further, no second harmonic was observed in LC with the vertically oriented molecules from the horizontally polarized pump. It allows us to conclude that d_{22} and d_{32} of the LC nonlinear tensor are close to zero. In contrast, the SHG efficiency in LC from the vertically polarized pump is comparable with the SHG efficiency in LN. From this measurement, we retrieved d_{23} and d_{33} of the LC nonlinear tensor by fitting the data with

$$\eta_{SHG} = \frac{(d_{23}\cos\phi_A + d_{33}\sin\phi_A)^2}{n_{LC}^2(\omega_P) n_{LC}(2\omega_P)}, \tag{S10}$$

with the values $d_{23} = 0.05 \pm 0.02$ (assumed to be equal to zero) and $d_{33} = 1.43 \pm 0.02$.

Next, we fixed the polarization of the detected second harmonic (horizontally or vertically) and changed the polarization of the pump. Again, we measured the SHG efficiency in LN as a reference (red points in Extended Data Fig. 3b). We observed no horizontally polarized second

harmonic from LC (green points in Extended Data Fig. 3b), while the vertically polarized second harmonic is quite strong. From the fit of the data (blue points in Extended Data Fig. 3b) with the function

$$\eta_{SHG} = \frac{\left(d_{32}\cos^2\phi_P + d_{33}\sin^2\phi_P + d_{34}\sin 2\phi_P\right)^2}{n_{LC}^2(\omega_P)n_{LC}(2\omega_P)}, \tag{S11}$$

we extracted the near-zero values for d_{32} and d_{34}, and the similar estimation for $d_{33} = 1.46 \pm 0.02$. Therefore, after the comparison of the measured values of the LC nonlinear tensor with the reference values of that for LN, we concluded that only one component of the d tensor of LC is non-zero, $d_{33} \approx 20\,\frac{\mathrm{pm}}{\mathrm{V}}$.

Further on, we illustrate the effect of molecular orientation switching under the applied electric field by measuring the second harmonic radiation as a function of the applied voltage (Extended Data Fig. 4, a and b). We tested the sample with the π molecular twist pumped at $850\,\mathrm{nm}$ (OPOTEK Opolette 355, $5\,\mathrm{ns}$ pulse duration, $20\,\mathrm{Hz}$ repetition rate). The energy of the pulses was typically in the range between $50\text{-}100\,\mu\mathrm{J}$. A longpass filter with the edge at $800\,\mathrm{nm}$ was used to filter out any SHG signal generated in the OPO. The pump beam was focused on the sample through a 50/50 beamsplitter and 10x, 0.3 NA objective (Nikon), which was also used to collect the reflected light consisting of both reflected laser light and generated SHG. A shortpass filter at $550\,\mathrm{nm}$ rejected the reflected laser light. The collected light was analyzed by an imaging spectrometer (Andor Shamrock SR-500i) with a $15\,\mu\mathrm{m}$ wide slit, a grating with 300 lines per mm, and a CCD detector with a resolution of 1600 pixels. Typical exposure times were $0.5\text{-}2\,\mathrm{s}$. By observing the SH signal as a function of time and switching on the voltage, we infer that the response time is 0.5s (Extended Data Fig. 4c).

3 POSSIBLE EFFECTS OF THE PUMP BEAM ON THE SAMPLE AND OTHER NONLINEAR EFFECTS.

Throughout the measurements, no influence of the excitation laser on the sample structure was observed. In the bright-field and crossed polarisation microscopy images we do not observe any changes on the sample (both momentary or permanent) caused by the pump beam. Even several hours of illumination with a few mW or using the sample continuously for a couple of months did not cause any visible permanent changes. Damage threshold testing has shown that only powers above $60\,\mathrm{mW}$ and illumination times of more than $30\,\mathrm{min}$ caused a small permanent damage spot. Other effects, such as optical Fréedericksz transition or phase transitions due to heating, were not observed either. According to literature, these effects are typically observed in liquid crystals at several tenths of milliwatts of laser power for a diffraction-limited spot through a high NA objective [4]. In addition, the refractive index (and birefringence) of FNLCs change relatively little with the temperature [5]. Lastly, no other nonlinear effects, such as four-wave mixing, stimulated orientational scattering, or photorefractivity, were observed, which is

in accordance with previous studies where higher powers or pulsed excitations were necessary [6, 7].

4 MAXIMUM-LIKELIHOOD METHOD.

Since a density matrix must be Hermitian, it can be represented as a product of two Hermitian-conjugate matrices,

$$\hat{\rho} = \frac{\hat{T}^{\dagger}\left(\vec{t}\right)\hat{T}\left(\vec{t}\right)}{Tr\left(\hat{T}^{\dagger}\left(\vec{t}\right)\hat{T}\left(\vec{t}\right)\right)}, \tag{S12}$$

where $\hat{T}\left(\vec{t}\right)$ is a semi-diagonal matrix given as a function of a real-valued vector $\vec{t}$,

$$\hat{T}\left(\vec{t}\right) = \begin{pmatrix} t_1 & t_4 + \imath t_5 & t_8 + \imath t_9 \\ 0 & t_2 & t_6 + \imath t_7 \\ 0 & 0 & t_3 \end{pmatrix}, \ \vec{t} \in \mathbb{R}^9. \tag{S13}$$

We can write the elements of the density matrix as a function of parameters $\vec{t}$ explicitly as

$$\begin{cases} \rho_{11} & = t_1^2, \\ \rho_{12} & = t_1 t_4 + \imath t_1 t_5, \\ \rho_{13} & = t_1 t_8 + \imath t_1 t_9, \\ \rho_{22} & = t_2^2 + t_4^2 + t_5^2, \\ \rho_{23} & = t_2 t_6 + t_4 t_8 + t_5 t_9 + \imath\left(t_2 t_7 + t_4 t_9 - t_5 t_8\right), \\ \rho_{33} & = t_3^2 + t_6^2 + t_7^2 + t_8^2 + t_9^2, \end{cases} \tag{S14}$$

from which we can obtain the system of equations to find parameters $\vec{t}$,

$$\begin{cases} t_1 = \sqrt{\rho_{11}}, \\ t_4 = \Re\left(\rho_{12}\right)/t_1, \\ t_5 = \Im\left(\rho_{12}\right)/t_1, \\ t_8 = \Re\left(\rho_{13}\right)/t_1, \\ t_9 = \Im\left(\rho_{13}\right)/t_1, \\ t_2 = \sqrt{\rho_{22} - t_4^2 - t_5^2}, \\ t_6 = \left(\Re\left(\rho_{23}\right) - t_4 t_8 - t_5 t_9\right)/t_2, \\ t_7 = \left(\Im\left(\rho_{23}\right) - t_4 t_9 + t_5 t_8\right)/t_2, \\ t_3 = \sqrt{\rho_{33} - t_6^2 - t_7^2 - t_8^2 - t_9^2}. \end{cases} \tag{S15}$$

Although system (S15) has only one non-trivial solution, not all parameters t_i might be purely real if the experimentally retrieved values of the density matrix ρ_{ij}^{exp} are substituted into the

system, meaning that the experimental density matrix is not physical. For the MaxLi method, we took the real part of the solution of system (S15) as the initial guess. Then, the best fit of the density matrix is considered to be $\hat{\rho}\left(\vec{t}_{opt}\right)$ that has the form (S13) and gives the minimum deviation from the experimentally retrieved density matrix,

$$F\left(\vec{t}\right) = \sum_{i=1}^{3}\sum_{j\geq i}\left|\rho_{ij}^{exp} - \rho_{ij}\left(\vec{t}\right)\right|^2, \tag{S16}$$

$$F\left(\vec{t}_{opt}\right) = \min_{\vec{t}\in\mathbb{R}^9} F\left(\vec{t}\right). \tag{S17}$$

5 References

[1] Boyd, R. W. Chapter 1 - the nonlinear optical susceptibility. In *Nonlinear Optics (Third Edition)*, 1–67 (Academic Press, Burlington, 2008).

[2] Shoji, I., Kondo, T., Kitamoto, A., Shirane, M. & Ito, R. Absolute scale of second-order nonlinear-optical coefficients. *J. Opt. Soc. Am. B* **14**, 2268–2294 (1997).

[3] Dmitriev, V. G., Gurzadyan, G. G. & Nikogosyan, D. N. *Properties of Nonlinear Optical Crystals*, 67–288 (Springer Berlin Heidelberg, Berlin, Heidelberg, 1999).

[4] Škarabot, M. *et al.* Two-dimensional dipolar nematic colloidal crystals. *Phys. Rev. E* **76**, 051406 (2007).

[5] Kumari, P., Basnet, B., Lavrentovich, M. O. & Lavrentovich, O. D. Chiral ground states of ferroelectric liquid crystals. *arXiv preprint arXiv:2401.09675* (2024).

[6] Khoo, I. C. & Liang, Y. Stimulated orientational and thermal scatterings and self-starting optical phase conjugation with nematic liquid crystals. *Phys. Rev. E* **62**, 6722–6733 (2000).

[7] Sasaki, T., Katsuragi, A., Mochizuki, O. & Nakazawa, Y. Influence of the properties of ferroelectric liquid crystals on the spontaneous polarization reorientation photorefractive effect. *The Journal of Physical Chemistry B* **107**, 7659–7665 (2003).
